# Elephant Endotheliotropic Herpesvirus 1, 4 and 5 in China: Occurrence in Multiple Sample Types and Implications for Wild and Captive Population Surveillance

**DOI:** 10.3390/v14020411

**Published:** 2022-02-17

**Authors:** Nian Yang, Mingwei Bao, Biru Zhu, Qingzhong Shen, Xianming Guo, Wenwen Li, Ruchun Tang, Di Zhu, Yinpu Tang, David N. Phalen, Li Zhang

**Affiliations:** 1 Key Laboratory for Biodiversity and Ecological Engineering of Ministry of Education, Department of Ecology, College of Life Sciences, Beijing Normal University, Beijing 100875, China; chanynad@163.com (N.Y.); biruzhu@bnu.edu.cn (B.Z.); ruchun.tang@mail.bnu.edu.cn (R.T.); 201931200039@mail.bnu.edu.cn (D.Z.); typelephant@126.com (Y.T.); 2 Wild Elephant Valley, Jinghong 666100, China; baomingwei-11@163.com; 3 Ecotourism Management Institute of Yunnan Xishuangbanna National Nature Reserve, Jinghong 666100, China; sqz1196@163.com; 4 Research Institute of Xishuangbanna National Nature Reserve, Jinghong 666100, China; 2138519g@163.com; 5 Key Laboratory of Coastal Science and Integrated Management, First Institute of Oceanography, Ministry of Natural Resources, Qingdao 266061, China; liww1208@163.com; 6 Sydney School of Veterinary Science, Faculty of Science, University of Sydney, Sydney, NSW 2006, Australia; 7 Schubot Exotic Bird Health Center, College of Veterinary Medicine and Biomedical Sciences, Texas A&M University, 701 Farm to Market 2818 Service Road, College Station, TX 77840, USA

**Keywords:** Asian elephants, elephant endotheliotropic herpesviruses (EEHV), China, *Elephas maximus*, non-invasive samples

## Abstract

Elephant endotheliotropic herpesviruses (EEHVs) are important causes of death in both captive and wild Asian elephants (*Elephas maximus*). Nothing is known about the prevalence of EEHVs in wild or domestic elephants in China. To determine if EEHVs are present in elephants in China, 126 wild elephants from three populations and 202 captive individuals from zoos (*n* = 155) and the Wild Elephant Valley (*n* = 47) were screened using semi-nested polymerase chain reaction assays with EEHV-redundant and EEHV1/4/5-specific primers. EEHV1B and EEHV4 were detected in samples from both wild (EEHV1B:8/126; EEHV4:2/126) and captive (EEHV1B:5/155; EEHV4:9/155) elephants, while EEHV1A (six cases) and EEHV5 (one case) were only present in the captive elephants from the Wild Elephant Valley. EEHV1 was detected in blood and trunk and oral swabs; EEHV4 was detected in trunk and oral swabs as well as feces; EEHV5 was found in trunk and oral swabs. No significant age or sex association with EEHV1A, EEHV1B, or EEHV5 positivity was observed. An age association with EEHV4 positivity was found, with all unweaned elephants being EEHV4 positive, but an association with the sex of the elephant was not observed. These findings represent the first documentation of EEHV presence in captive and wild elephants in China. These findings also document EEHV1B and EEHV4 shedding in feces and demonstrate the utility of fecal screening as a tool for investigating EEHV4 infection in wild populations of elephants. It is recommended that EEHV testing be included in surveillance programs for captive and wild elephants in China.

## 1. Introduction

The elephant endotheliotropic herpesvirus (EEHV) poses a significant threat to the endangered Asian elephant (*Elephas maximus*) in captivity and in the wild [1,2,3,4]. Seven types (i.e., species) with a total of twelve subtypes of EEHV (EEHV1–7) have been identified, among which EEHV1A, 1B, 4, and 5 are endemic within Asian elephant populations [5,6,7,8]. Infections with EEHVs, in most cases, result in a subclinical but likely lifelong infection with intermittent virus shedding. However, infection in young Asian elephants is more likely to result in acute, fatal hemorrhagic diseases (EEHV-HD), most cases occur in elephants less than four years of age, and the vast majority occur in elephants less than eight years of age [8]. In fact, since its discovery in the 1990s, EEHVs have been documented to be the major cause of Asian elephant calf deaths in captivity [9,10,11,12]. EEHV also appears to be widespread in wild Asian elephants across their range, and it has been detected in elephant populations in India, Thailand, Laos, Myanmar, and Malaysia [1,2,3,13,14,15]. However, most positive cases of EEHV in wild elephants are detected in animals that were sick or dead and, therefore, the EEHV prevalence among wild Asian elephant populations is unknown [15,16].

Sub-clinically, infected elephants have been detected using molecular methods that detect viral DNA in oral [17] and trunk swabs, trunk flushes [17], and blood [17,18,19]. Weekly testing of blood samples for viremia is currently recommended for elephants under 8 years of age [20] to detect infections that might subsequently result in disease. Detecting virus by molecular methods is currently the only way to confirm the type and subtype of virus infecting an elephant, although new serological techniques may be able to differentiate between serologically distinct types in the future [21]. Both molecular and serological methods have been used to determine the prevalence of EEHV infection in elephant populations. Screening blood samples with molecular techniques from captive Asian elephant populations has been used to determine the prevalence of infection in them, and a prevalence range of 1.2–5.6% has been found [15,19]. However, these values are likely to significantly underestimate the actual prevalence of active and latent infections in these populations, as viremia is intermittent [22,23,24] and virus shedding may occur in the absence of viremia [21] and it, too, can be intermittent [21,25]. Additionally, large-scale serological studies of captive Asian elephants in zoos [23] and in their countries of origins [15,26] have found high prevalence of seropositivity in these populations ranging from 42.3% [26] and 43.9% [15] in Asian elephants in Thailand to 80% seropositivity in elephants in zoo collections [23]. Two studies using antigens produced in eukaryotic systems and, therefore, more likely to reflect the epitopes expressed on EEHVs [21,27] found 100% seropositivity in mature zoo elephants in the USA [21], Europe [27], and captive elephants in Laos [27], suggesting that some or all EEHVs are expected to infect all adult Indian elephants.

China has a population of approximately 300 wild Asian elephants. They are distributed among four geographically isolated populations in Yunnan Province: the Nangunhe, Pu’er-Mengyang, Mengla, and Shangyong populations, which have been isolated from each other for at least 20 years, and the Nangunhe population has been isolated from the other three populations for a least 50 years [28]. Individuals from the Shangyong and Mengla populations may cross the border into Laos, while the other two populations’ ranges are entirely within China. An additional approximately 320 captive Asian elephants are scattered among dozens of Chinese zoological collections. Wild elephant populations in China have been increasing in recent years; however, with increasing numbers of elephant calves being born, there has also been an increase in the number of dead and abandoned sick calves that have been found. The role that EEHVs play in these calves’ deaths and illnesses is not known, neither is the distribution and prevalence of EEHVs in the wild populations in general. Similarly, there have been no studies on the distribution and prevalence of EEHVs in captive elephants in China, and only a few institutions are aware of the dangers that EEHVs might pose to their collections.

Current molecular EEHV testing protocols in live elephants require that blood, trunk wash samples, or oral swabs be obtained [5,17,20,29,30], and enzyme-linked immunoassays for antibodies against EEHVs also require blood samples [21,26,27]. This means that only trained captive elephants can be safely sampled, and testing wild live elephants is not possible. However, in recent studies, EEHV2 DNA was detected in one of 70 fecal samples from African elephants (*Loxodonta africana*) [30], and EEHV antigen was detected in epithelial cells lining the gastrointestinal tract in Asian elephants infected with EEHV4 [31,32], suggesting that one or more EEHVs may be detected noninvasively in elephant feces [31,33].

The first objective of this this study was to determine if EEHVs were present in wild and captive Asian elephants in China. The second objective was to determine if EEHVs could be detected in elephants by screening feces for viral DNA and to compare the sensitivity of fecal screening with the traditional methods of blood and trunk and oral swab screening. Finally, based on the findings of this study, recommendations for EEHV surveillance and management among captive and wild Asian elephant populations in China are discussed.

## 2. Materials and Methods

### 2.1. Animal Ethics

All the samples collected in this study were for scientific purposes. Ethical approval to conduct this study was obtained from Ethic and Animal Welfare Committee, College of Life Science, Beijing Normal University. The approval number was CLS-EAW-2020-015.

### 2.2. Investigation of EEHV Presence in China 

#### 2.2.1. Sample Collection 

Four hundred and fifty-nine samples from wild (*n* = 126) and captive (*n* = 202) Asian elephants in China were collected from 2017 to 2020 (Table 1). All sampled elephants were clinically healthy, and their fecal forms were normal.

- Captive Populations

Samples were collected from 202 captive Asian elephants from 2018 to 2020, representing approximately 63% of the entire population of captive elephants in China. Samples were collected from two sources: elephants from the Wild Elephant Valley (*n* = 47) and elephants in traditional zoos (*n* = 155) (Table 1). 

**Figure 1:**
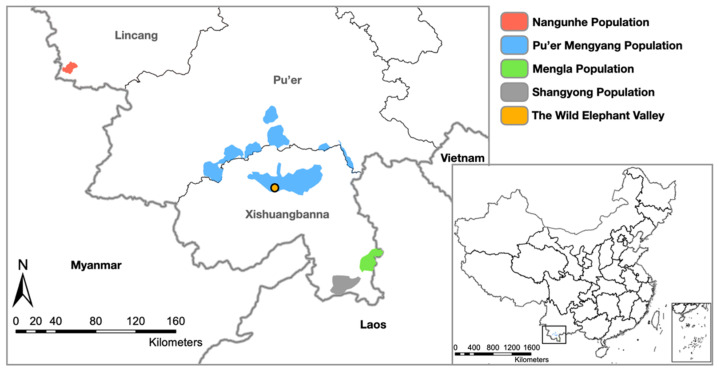
Distribution of wild Asian elephant populations in China and the location of the Wild Elephant Valley.

The Wild Elephant Valley (100°51’33.2172”, 22°10’39.3636”) facility is located within the Pu’er-Mengyang Asian elephant population area (Figure 1). It is a tourist attraction and contains an Asian elephant rescue center. It has a mixture of imported elephants that are occasionally moved between institutions as well as captive-bred and rescued elephants. The elephants in this facility are allowed into their natural environment to forage and roam during the day accompanied by their mahouts; thus, they cannot have active direct contact with wild elephants during this time. However, during the night, wild elephants may visit their enclosure, and Wild Elephant Valley elephants can have physical contact with wild elephants through the bars of their enclosures; therefore, the two populations are not fully isolated. 

The origin, age, and sex of Wild Elephant Valley elephants and zoo elephants were recorded based on clinical records, and the elephants were categorized into groups according to their age. The age groups were based on Arivazhagan and Sukumar [34] with minor modification: adult (>15 y); sub-adult (5 < X ≤ 15 y); juvenile (3 < X ≤ 5 y); unweaned elephants (≤3 y).

The majority of Asian elephants in zoos were imported from other Asian elephant range countries with a small number of captive-bred elephants. These animals are routinely moved among zoos. Only fecal samples were taken from the zoo elephants (*n* = 155). 

Elephants were classified as herpesvirus virus positive if herpesvirus DNA was detected in one or more samples.

- Wild Populations

In 2017, fecal samples from three wild Asian elephant populations (i.e., Pu’er-Mengyang (*n* = 81), Mengla (*n* = 26), and Shangyong (*n* = 19)) were collected (Figure 1). A hundred and twenty-six individuals were identified by microsatellite markers [35], and their fecal samples were used in this study. The tested individuals were aged by bolus diameter according to the rearranged Von Bertalanffy growth equation: $t=\left(t_{0}-\frac{1}{k}\right)\times \left(\ln\left(1-\frac{L}{L_{\infty }}\right)\right)$; t is the mean age, t_0_ is the theoretical age at which an elephant’s bolus would have a length measurement of 0 if the same growth pattern was applied from birth to later life, K is the Brody growth coefficient, and L_∞_ is the theoretical maximum size of the bolus diameter [36,37]. Age groups were classified in the same way as captive elephants.

At the time of sampling, there was approximately 280 wild individuals in China. The approximate number of each sampled population is shown in Table 1.

#### 2.2.2. DNA Extraction

Fecal DNA was extracted using the QIAamp DNA Stool Mini Kit (Qiagen, Hilden, Germany), and DNA from blood and trunk and oral swabs was extracted using the DNeasy Blood & Tissue Kit (Qiagen, Germany) with a minor modification to the manufacturer’s protocols. The centrifugation time of the fecal samples after digestion was extended to 25 min to assure that all undigested material was pelleted. All DNA samples were stored at −20 °C until analysis.

#### 2.2.3. PCR Screening for EEHV DNA

EEHV DNA was detected using semi-nested PCR with redundant EEHV DNA polymerase (POL) primers and specific EEHV1/4/5 POL primer sets and EEHV1-viral G protein-coupled receptor (EEHV1-vGPCR) primer sets [29,38] (Appendix A) and Qiagen HotStarTaq Master Mix Kit (Qiagen, Germany). Samples were first tested by redundant EEHV POL and EEHV1, 4, and 5 POL primer sets for EEHV subtype identification. Then, the EEHV1 positives in the first screen were amplified by EEHV1-vGPCR primer sets for further phylogenetic analysis. 

The DNA amplifications were carried out on a Veriti 96-Well Thermal Cycler (Applied Biosystems, Singapore) using the following conditions: One cycle of 95 °C for 15 min, followed by 35 cycles of 94 °C for 30 s, 55 °C for 90 s, and 72 °C for 60 s; then, the samples were held at 72 °C for 5 min and immediately chilled at 4 °C. Two microliters of the solution containing the purified DNA was used in a 20 μL reaction. For the second and third round PCR reactions, 2 μL of the previous 20 μL PCR reaction was used. The amplification products were separated by electrophoresis on 1.5% agarose gels containing SYBR gel stain (Invitrogen, Waltham, MA, USA) and visualized under ultraviolet light. Positive samples were sent for sequencing (BGI, Beijing, China).

- Analytical sensitivity of the PCR assay

To determine the analytical sensitivity of the PCR assay, plasmids containing U38(POL) fragments of EEHV1, 3, and 4 were synthesized. The plasmid DNA was added into the EEHV-negative DNA in this study. The 10-fold serial dilutions of the synthesized plasmid DNA were created, making the final concentration of the plasmid DNA in the EEHV-negative DNA starting from 10^5^ to 10 copies/μL. The products were then amplified by the PCR assay employed in the paper.

- Inhibitor test for the fecal DNA samples

Given that a zoo elephant diet is different from that of wild elephants as well as the Wild Elephant Valley elephants and that a very low prevalence of EEHV DNA was detected in feces from zoo elephants, we sought to determine if it might contain inhibitors. The plasmid DNA used in the PCR sensitivity test was added to 105 EEHV-negative fecal DNA samples and amplified as described above. Of these, forty samples were from the Wild Elephant Valley (*n* = 44), forty-two DNA samples were from randomly selected zoo elephants (*n* = 155), and twenty-three DNA samples were from randomly selected wild elephants (*n* = 126). The final plasmid concentration in the PCR system was 500 copies/μL.

#### 2.2.4. Age and Sex Differences 

Information on the age composition and sex of elephants in the Wild Elephant Valley is well documented. Thus, the association between age or sex on EEHV infection of elephants in the Wild Elephant Valley was explored. Sex and age data (*n* = 47) for the elephants in the Wild Elephant Valley in 2020 were used in the analysis (Appendix B). Significant tests for associations between EEHV-positive elephants and the age and sex of the tested elephants were based on the Chi-squared test and Fisher’s exact test (*p* ≤ 0.05) [39,40].

#### 2.2.5. Phylogenetic Analysis

Sequences were analyzed in MEGAX and were compared with the EEHV sequences in GenBank using NCBI BLAST. For the EEHV1 analysis, a vGPCR (U51) locus was used. All of the U51 fragments of EEHV1 in the NCBI database [41] were downloaded and aligned in MEGAX [42] with the sequences found in this study. Aligned sequences were then trimmed and translated into protein sequences. Two EEHV6 and one EEHV2 vGPCR sequences were added to the analysis. Identical sequences were then removed using the DISTANCE tool, and one representative sequence was retained for each set of identical sequences. The result was used to construct a neighbor-net by ProteinMLdist using SplitsTree4 [43], and the bootstrap value was obtained using 1000 replicate bootstrapping. The information on the country of discovery for all the sequences involved in the analysis was summarized in a bar chart (Numbers, 2021, Apple). According to Long, Latimer, and Hayward [8] and Sripiboon et al. [44], the delineation of clusters is based on amino-acid polymorphisms for the U51 (vGPCR) protein. We analyzed the cluster distribution, and then used Keynote (Keynote, 2021, Apple) to annotate the neighbor-net and integrated it with the bar chart into one figure.

For the EEHV4 and 5 phylogenetic analyses, the DNA POL(U38) locus was used. All the U38 sequences of EEHV2/3/4/5/6/7 in NCBI were downloaded and aligned with ClustalW in MEGAX with the sequences found in this study [41,42], trimmed, and translated into protein sequences. Two EEHV1 U38 sequences were added to the analysis. The identical sequences were then removed using the DISTANCE tool, and one representative sequence was retained for each set of identical sequences. The best model for the maximum likelihood tree was selected according to the MODELS in the MEGAX recommendation. Jones Taylor Thornton Model plus Gamma Distributed (JTT + G) was utilized for EEHV4 and EEHV5 U38 locus maximum likelihood tree construction. The bootstrap value was obtained using 1000 replicate bootstrapping.

## 3. Results

### 3.1. Number of EEHV-Infected Elephants and EEHVs Detected

#### 3.1.1. Detection of EEHV in Different Sample Types of Wild Elephant Valley Elephants

A comparison of the PCR results for the feces, blood, and trunk and oral swabs from 47 valley elephants is shown in Table 2. Overall, fourteen elephants (29.8%; 14/47) were positive for EEHVs. Positive cases were detected in imported, rescued elephants, and in captive breeding elephants. Nine of the elephants were infected with a single EEHV including two EEHV1A, two EEHV1B, four EEHV4, and one EEHV5. Co-infections were found in five elephants (EEHV1A/EEHV4, *n* = 4; EEHV1B/EEHV4, *n* = 1). 

Only EEHV4 was detected in feces. EEHV1A and EEHV1B were detected in blood, and EEHV1A, EEHV1B, EEHV4, and EEHV5 were detected in both trunk and oral swabs. Four of the five co-infected elephants showed PCR positivity for two samples, each detected a separate EEHV type/subtype, while one elephant had three PCR-positive samples: two samples detected EEHV1 and another sample showed EEHV4 positivity. Excluding co-infected elephants, five elephants with two sample types showed PCR positivity simultaneously, and one elephant with three sample types showed PCR positivity at the same time.

A total of nine EEHV1 positive cases (19.1%; 9/47) were found in blood and trunk and oral swabs, six of which were EEHV1A (12.8%; 6/47) and three of which were EEHV1B (6.4%; 3/47). No EEHV1 positivity was detected in fecal samples. The blood sample from one EEHV1A-positive elephant was not collected, and three EEHV1A-positive cases were detected in blood samples (60%; 3/5). Three EEHV1A positives were found in trunk swabs (50%; 3/6), and one case was detected in oral swabs (16.7%; 1/6). Among them, one (V15) was positive for EEHV1A in blood and trunk swabs. The remaining four cases were all positive for one single sample type. For EEHV1B, two positives were found in blood samples (50%; 2/4), and the other positive elephant (V43) was positive in both trunk (25%; 1/4) and oral swabs (25%; 1/4). 

A total of nine cases of EEHV4 positivity (19.1%; 9/47) were detected in feces and trunk and oral swabs, and no EEHV4 positivity was found in the blood. The fecal samples from two EEHV4-positive elephants were not collected, and four EEHV4-positive elephant fecal samples collected were detected positive (57.1%; 4/7). Three cases (33.3%; 3/9) were detected in trunk swabs, and six (66.7%; 6/9) were detected in oral swabs. Among them, one (V36) was positive for EEHV4 in feces and trunk and oral swabs; one (V38) was positive in feces and oral swabs; one (V39) was positive in feces and trunk swabs. The remaining six cases were all positive for a single sample type.

One EEHV5-positive elephant (V10) (2.1%; 1/47) was identified in the tests, and it was positive for both trunk (50%; 1/2) and oral swabs (50%; 1/2).

#### 3.1.2. Detection of EEHV in Fecal Samples from Captive and Wild Elephants

The screening results for EEHVs in the fecal samples from captive and wild elephants are shown (Table 3). This sub-study includes fecal sample testing results in the Wild Elephant Valley from the previous sub-study, thus comparing the presence of EEHV in feces in different Asian elephant populations in China. A total of 16 EEHV infections from 16 elephants were detected in the 325 wild and captive elephants screened with a total prevalence of 4.92%. Two EEHVs were detected: EEHV1B, and EEHV4.

- Wild Populations

A total of ten wild elephants were found to be infected with an EEHV (7.94%; 10/126). There were eight EEHV1B (6.35%: 8/126) and two EEHV4 (1.59%; 2/126) positive elephants, and EEHV1A was not detected. According to the fecal bolus diameters, seven EEHV1B-positive elephants and all EEHV4-positive elephants were sub-adults between 5 and 15 years old. A single EEHV1B-positive elephant was an adult. EEHV1B was detected in the population inside China (i.e., Pu’er-Mengyang) and the populations traveling between China and Laos (i.e., Shangyong and Mengla). The two EEHV4 positives were detected from two elephants in the Mengla population.

- Captive populations

Two EEHV1B positives (1.01%; 2/199) and four EEHV4 positives (2.01%; 4/199) were detected from the 199 captive elephants tested. EEHV1A and EEHV5 were not detected in this cohort. The two EEHV1B-positive zoo elephants (1.29%; 2/155) were housed in separate facilities and had never been in contact. The four EEHV4-positive elephants (9.09%; 4/44) were all from the Wild Elephant Valley.

- Analytical sensitivity of the PCR assay and prevalence of inhibitor samples from the Wild Elephant Valley, zoo, and wild elephants

All PCR assays were able to detect a hundred copies and occasionally (2/16 times) detected ten copies. Two of the forty Wild Elephant Valley samples (5.00%; 2/40) contained inhibitors, five of the forty-two zoo samples (11.90%; 5/42) contained inhibitors, and none of the twenty-three wild samples (0.00%; 0/23) tested contained inhibitors.

### 3.2. Age and Sex Differences

**Figure 2:**
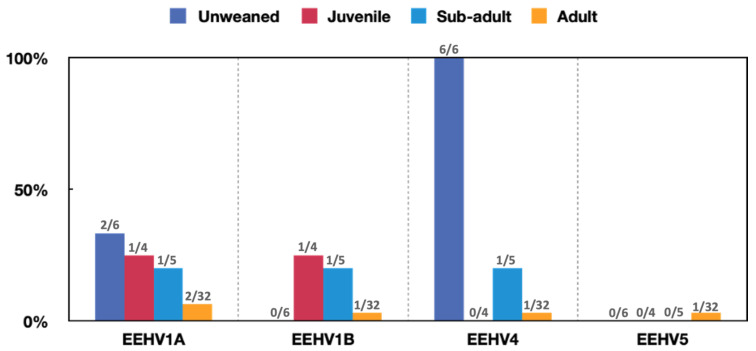
EEHV positivity rate in the different age groups in the Wild Elephant Valley. (There were 6 unweaned elephants, 4 juvenile elephants, 5 sub-adults, and 32 adults in the Wild Elephant Valley).

The positivity rate for the age groups of each virus in the Wild Elephant Valley is shown in Figure 2. There were no significant association between EEHV1A, 1B, or 5 positivity and age (*p* = 0.234 > 0.05; *p* = 0.183 > 0.05; *p* = 0.921 > 0.05); however, a significant association between EEHV4 positivity and age was observed (*p* = 0.000 < 0.05). Further pairwise comparison of the EEHV4-positive rate between age groups showed that there were significant differences in infection rates between the non-weaned group and the subadults, as well as between the non-weaned group and the adult group (*p* = 0.005 < 0.05; *p* = 0.000 < 0.05). An association between EEHV1A, EEHV1B, and EEHV4 infection rate and sex was not detected (*p* = 0.947 > 0.05; *p* = 0.726 > 0.05; *p* = 0.106 > 0.05; *p* = 0.844 > 0.05).

### 3.3. Phylogenetic Analysis

We analyzed the amplification results of vGPCR locus of all EEHV1-positive samples and the POL locus of EEHV4 and EEHV5 positive samples obtained in this study. Only the POL fragment of samples from one elephant (V15) of the Wild Elephant Valley was amplified, and the amplification of vGPCR fragments failed. Therefore, this elephant (V15) was not included in the analysis. 

As shown in Table 4, a total of seven strains (CN01–CN07) were identified, of which EEHV1A contained three strains (CN01–CN03), EEHV1B contained two strains (CN04–CN05), and EEHV4 and EEHV5 each had one strain (i.e., CN06 and CN07). CN01 and CN05 were novel strains, different from those found in other countries. The other five strains were identical to the strains found in Asian elephants in Thailand and North America (Table 4). 

**Figure 3:**
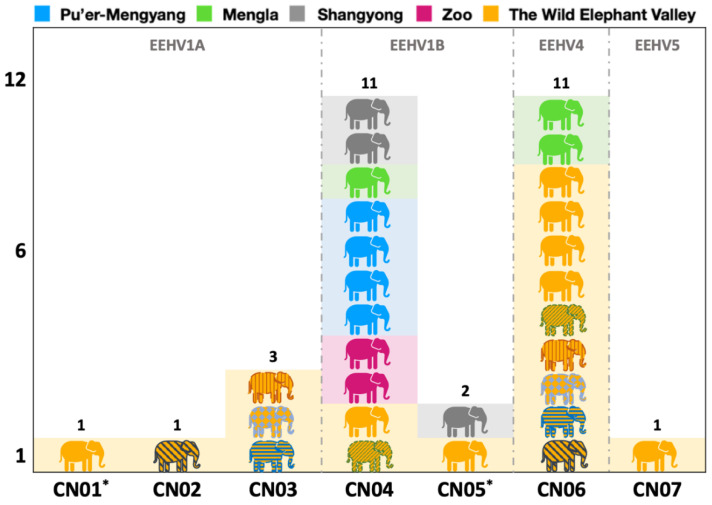
Distribution of each EEHV strain found in this study in each population. CN01* and CN05* were novel strains detected in this study. Elephant shapes with patterns indicate five EEHV1/EEHV4 co-infected elephants in the Wild Elephant Valley, and identical patterns refer to the same elephant.

The distribution of each EEHV strain found in this study for each population is shown in Figure 3. Seven strains were present in the Wild Elephant Valley area, and four were present only in the Wild Elephant Valley population (CN01–CN03 and CN07). The other three strains were also detected in other populations, and only 1–2 strains were present in each population. Strain CN04 was distributed in all populations tested. In addition to strain CN04, CN06 was present in the wild elephant population in Mengla and CN05 in the wild elephant population in Shangyong, while only CN04 was present in the zoo elephant population and wild elephants from Pu’er-Mengyang. In addition, CN04 and CN05 were found in both wild and captive populations. 

**Figure 4:**
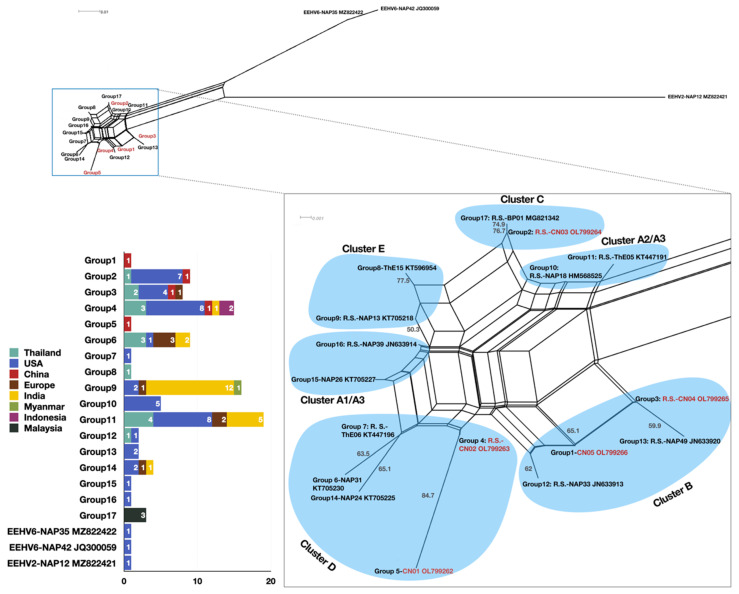
Neighbor-net constructed based on the vGPCR/U51 locus from ninety-eight EEHV1, one EEHV2, and two EEHV6 cases using the ProteinMLdist by SplitsTree4 based on 159 amino acid sites, with bootstrap values indicated and the composition of the geographical distribution of the strain in each group. (The red group in the net contains the strains found in this study. R.S. = representative strain).

Genetic analysis was conducted to identify the variation and discover the relatedness between the isolates found in our study and other studies. For the EEHV1-positive samples, based on 159 amino acid sequences, the neighbor-net showed that the five strains in this study plus the 93 EEHV1 vGPCR (U51) sequences belonging to different individuals found in the NCBI database belonged to a total of 17 groups (Figure 4). The strains found in China were concentrated in Cluster B, C, and D with two strains each in Cluster B and D and one strain in Cluster C. CN01 and CN02 were in Group 5 and Group 4 in Cluster D, and Group 4 also contained strains from Thailand, North America, India, and Indonesia. CN03 was in Group 2 in Cluster C, and Group 2 also contained strains from Thailand and North America. CN04 and CN05 were in Group 3 and Group 1 in Cluster B, and Group 3 also contained strains from Thailand, North America, and Europe.

**Figure 5:**
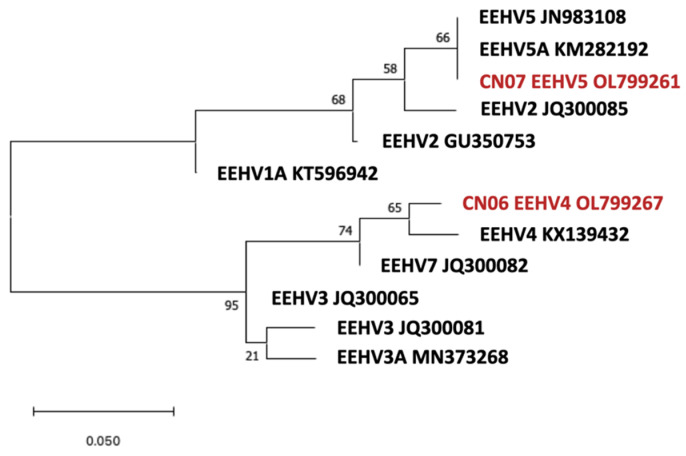
Genetic relationships between EEHV4 (CN06) and EEHV5 (CN07) detected in this study and other identified EEHVs. This phylogenetic tree was analyzed by the maximum likelihood method based on 63 amino acid sites of EEHV DNA polymerase(U38) with bootstrap values indicated. (The strains in red are strains found in this study, and the black strains are reference strains).

The maximum likelihood phylogenetic tree constructed based on the POL(U38) gene locus of the EEHV4 and EEHV5 strains found in this study and EEHV1–5 and EEHV7 found in other studies is shown in Figure 5. Based on the 63 amino acid sequences, the EEHV4 and EEHV5 genomes were well conserved, and the CN06 and the reference EEHV4 POL genome (KX139432) found in Thailand only had 1.5% differences, and CN07 was identical to the reference EEHV5 genomes. 

## 4. Discussion 

The risks posed by the EEHVs to captive and wild Asian elephants are widely recognized. Surveys of captive, semi-captive, and, to a lesser extent, wild elephants have been conducted in several countries, and surveillance for captive and semi-captive elephants have been developed to suit local conditions [2,14,15]. China is an Asian elephant range country and has a captive Asian elephant population; however, the EEHV status of China’s wild and captive elephants is not known. 

### 4.1. Types, Subtypes, and Strains Identified and the Potential Implications for the Occurrence of EEHV-HD

This large-scale study reports the findings of the first survey for the presence of EEHV in captive and wild Asian elephants in China. In this survey, all EEHV types and subtypes (EEHV1A, 1B, 4, and 5) found in Asian elephants elsewhere were found in captive Asian elephants and fewer types (EEHV1B and 4) were found in the wild populations. Most of the EEHV strains identified had been previously documented in elephant populations outside of China; however, novel EEHV1A and EEHV1B strains are described here for the first time. Sequences from the EEHV1A and EEHV1B genetic Groups 6–17, which contained the majority of sequences from Asian elephants sampled in India, were not found in the Chinese populations. Additional testing of elephants in China will be required to determine if these findings represent geographical trends in EEHV1A and 1B distributions or if they are the result of overall low sample sizes of sequenced EEHV viruses.

EEHV-HD is caused predominately by strains of EEHV1 but occasionally also by EEHV4 and EEHV5 [5,7,8,38]. Therefore, finding these strains in the captive population of elephants held at the Wild Elephant Valley facility suggests that calves born at these facilities are at risk of developing EEHV-HD. In addition, given that elephants are routinely moved among institutions in China, including the Wild Elephant Valley facility, it is likely that these strains of EEHV are present in other captive collections of elephants as well and pose the same threat to calves in their collection. Contact between wild elephants and captive elephants at the Wild Elephant Valley facility, although minimal and restricted, may allow EEHV strains present in the captive population to spread to wild elephants in this part of China.

### 4.2. Using Multiple Sampling Sites Improved the Detection Rate of EEHV Infections and Types

This is only one of a few studies where it was possible to compare the findings of PCR screening of blood, oral swabs, and trunk swabs for EEHV DNA in the same elephant, and this is the first study where the results of the PCR screening of these samples were compared to the PCR screening of DNA from elephant feces. Our results show, as has been noted previously, that if PCR screening is to be used as a method to detect an active EEHV infection, that screening samples from trunk swabs, oral swabs, and blood will detect more active infections then either one alone and will be more likely to detect infections with multiple EEHV types. Combining the results of testing fecal DNA with testing of one or all three other samples also improved the chance of detecting active virus infections in the elephants and detecting infections with multiple genotypes in this study. Therefore, we recommend that future investigations and routine screening protocols for active EEHV infections in elephants include testing samples from blood, oral swabs, trunk swabs, and fecal DNA whenever possible.

We chose to screen elephants for active EEHV infection by screening fecal DNA for EEHV DNA, because if EEHV DNA could be detected in this sample, it would mean that the presence of EEHV types and possibly even the prevalence of active EEHV infections in wild elephants could be determined, and collecting a fecal sample would be easier and safer than collecting blood and oral and trunk swabs from captive elephants [30,33]. Our results show that active EEHV infections can be detected in elephants by screening fecal DNA. However, a single fecal sample, as with samples from other sites, did not detect all active EEHV infections, and not all EEHV types were detected in fecal DNA [31,33]. While our data set was relatively small, it provides proof that screening of a single fecal DNA sample will be able to detect some, by far not all, elephants actively infected with EEHV1B and EEHV4, and given that EEHV1A DNA was never detected in an elephant fecal sample, it may not be able to detect elephants with active EEHV1A infections. Therefore, as discussed above, when testing elephants for EEHV1A, 1B, and 4 active infections, the chances of detecting them are improved by testing a combination of oral and trunk swabs and blood in conjunction with feces. However, testing feces can provide valuable information about the presence or absence of EEHV1B and EEHV4 types in populations where blood and oral and trunk swabs cannot be obtained. The value of screening fecal DNA to detect active EEHV5 infections in Asian elephants could not be determined by this study as only one elephant with an active EEHV5 infection was identified and EEHV5 DNA was not detected in its fecal DNA. 

### 4.3. Impact of Age on Prevalence of Active EEHV Infections

Our findings are similar to a previous study that showed that viremia and virus shedding of elephants infected with EEHV1A or 1B is most common in juvenile elephants between 1 and 5 years of age [19]. Comparable studies of the effect of age on virus shedding in elephants infected with EEHV4 and 5 have not been conducted. However, our study suggests that younger elephants are also more likely to have active EEHV4 infections than older animals, as of the nine positive cases of EEHV4 in the Wild Elephant Valley, six were unweaned elephants. However, this may be a result of the sampling constraints. Further validation by future studies is needed.

### 4.4. Possible Explanations for Variations in Fecal EEHV Prevalence in the Three Elephant Populations Studied

The prevalence of EEHV PCR positive elephants, based on testing of fecal DNA screening, in Chinese zoological collections was low (1.29%) compared to the Wild Elephant Valley (9.09%) and wild populations (7.94%). The reason for this is not known but could reflect a higher percentage of samples containing inhibitors in the fecal samples from the zoo animals compared to those from the Wild Elephant Valley and from the wild elephants. It might also reflect the quality of the DNA in these samples, and though not tested, DNA degradation may have occurred more commonly during the sample collection and storage of the feces from the zoo elephants compared to the samples from the Wild Elephant Valley elephants and wild elephants. Including an internal control for DNA quality, such as testing for the elephant TNF gene, would address this question in future studies. 

### 4.5. Molecular versus Serological Screening of Asian Elephants for EEHV 

In this study, we used PCR assays to detect active infections in the elephants that we studied instead of screening for infection using serology, as the only population of elephants that we could obtain blood samples from was the captive population housed at the Wild Elephant Valley. Using molecular screening also allowed us to compare the sequences of the detected types and subtypes to those reported in other countries [15]. Screening elephants with PCR assays, however, has its limitations, as it only detects elephants with active infections and not latently infected elephants that are not viremic or shedding EEHV. 

In recent years, there have been great advances in the development of sensitive and specific serological assays that are able to identify elephants that are infected with EEHVs. These findings show that the prevalence of virus infection in the populations that have been tested using serology far exceeds that detected using molecular methods screening for viral DNA in blood or other samples [21,26,27]. In fact, it has been suggested that EEHV infections may be ubiquitous in Asian elephants greater than five years old [21,27]. It is therefore extremely likely that the overall prevalence of EEHV-positive elephants in the captive and wild populations that we studied is considerably higher than what we were able to detect. It is hoped that as soon as these serological assays are further refined and become more widely available, they may be able to be used for future studies and screening protocols in China, especially as they appear to be particularly useful for identifying naive elephant calves that would be at the highest risk for developing disease [21,27]. 

### 4.6. Implications of These Findings for Asian Elephants in China

Overall, there are relatively few captive Asian elephants in China, and it is difficult and costly to acquire new stock. Therefore, it is important to do everything possible to protect the health of the Asian elephants that are present in captive populations in China and ensure that as many elephant calves that are born to this population survive to reproduce. To protect calves born to captive elephants from EEHV disease, we propose that the screening recommendations of Luz and Howard [20] be followed at least in juvenile elephants. Based on these recommendations, we suggest elephants less than 8 years old should have blood samples and preferably trunk swab, oral swab, or and feces tested by PCR for EEHVs at weekly intervals and 2–4 times per year after that. Elephant calves that are found to be infected with an EEHV and found to have a rising virus titer or are experiencing signs consistent with EEHV disease should be treated with aggressive supportive care and an antiviral drug [45,46]. In addition, all sick and dead calves should be routinely tested for EEHV infection. In order to address the need for such testing and fill the vacancy of bodies in China that can perform official EEHV testing rather than research, it is recommended that a laboratory that is able to perform these assays be established and that they be a repository for data generated through this testing. Moreover, as antiviral drugs are costly and may not be easily sourced in an emergency situation where an elephant calf is stricken with disease caused by an EEHV, we recommend that enough antiviral medication to treat one or more calves be stockpiled in China and made available to captive elephant collections as needed.

The impact of EEHVs on the wild Asian elephant populations in China is unknown, but at present it seems to be minimal. The wild Asian elephant populations have maintained a strong annual growth rate of 3–5% in recent years as a result of aggressive conservation efforts [47]. However, as Asian elephant populations increase in size within the confines of the current sanctuaries, new stressors may arise impacting EEHV shedding and transmission [48], and dead wild elephant calves have been found. We therefore recommend testing tissue or blood samples for all dead wild elephants found during routine patrols or samples from live wild elephants obtained by chance. In addition, to obtain information on EEHV-related dynamics in wild elephant populations, we recommend conducting regular surveys for EEHV fecal shedding in these populations.

## Figures and Tables

**Table 1:** The number of samples collected for each sample type of each population, the total number of sampled individuals, and the estimated or actual population.

		Sampling Size Per Sample Type				Total Number of Sampled Individuals	Estimated or Actual Population
		Feces	Blood	Trunk Swab	Oral Swab		
Wild (*n* = 126)	Pu’er-Mengyang	81	NA	NA	NA	81	~140
	Mengla	26	NA	NA	NA	26	~45
	Shangyong	19	NA	NA	NA	19	~65
Captive (*n* = 202)	Zoo	155	NA	NA	NA	155	~270
	Wild Elephant Valley	44	40	47	47	47	47

**Table 2:** Age, sex, origin, sample types, and EEHV strains detected in EEHV-positive elephants in the Wild Elephant Valley.

		Age ^a^	Sex ^b^	Origin ^c^	Feces ^d^			Blood ^d^			Trunk Swab ^d^			Oral Swab ^d^		
					EEHV1	EEHV4	EEHV5	EEHV1	EEHV4	EEHV5	EEHV1	EEHV4	EEHV5	EEHV1	EEHV4	EEHV5
1	V10	30 y	M	TL	−	−	−	−	−	−	−	−	+	−	−	+
2	V15	21 y	M	TL	−	−	−	1A	−	−	1A	−	−	−	−	−
3	V31	14 y	M	R	−	−	−	1B	−	−	−	−	−	−	−	−
4	V33	5 y	M	R	−	−	−	1A	−	−	−	−	−	−	−	−
5	V34	4 y	F	Cb	NA	NA	NA	1B	−	−	−	−	−	−	−	−
6	V36	2 y	F	Cb	−	+	−	−	−	−	−	+	−	−	+	−
7	V37	2 y	F	Cb	−	+	−	1 A	−	−	−	−	−	−	−	−
8	V38	14 m	M	LAO	−	+	−	NA	NA	NA	−	−	−	−	+	−
9	V39	17 m	F	Cb	−	+	−	NA	NA	NA	−	+	−	−	−	−
10	V41	5 m	F	Cb	NA	NA	NA	NA	NA	NA	−	−	−	−	+	−
11	V42	2 m	F	Cb	NA	NA	NA	NA	NA	NA	1A	−	−	−	+	−
12	V43	36 y	F	MM	−	−	−	−	−	−	1B	−	−	1B	+	−
13	V44	23 y	F	MM	−	−	−	−	−	−	−	+	−	1A	−	−
14	V45	8 y	F	Cb	−	−	−	−	−	−	1A	−	−	−	+	−

^a^ y = years, m = months; ^b^ M = male, F = female; ^c^ TL = Thailand, R = rescued, Cb = captive bred, LAO = Laos, MM = Myanmar; ^d^ − = negative, NA = not available, + = positive, 1A = EEHV1A positive, 1B = EEHV1B positive.

**Table 3:** Age, sex, origin, and EEHV strains of the elephants that tested EEHV positive in fecal DNA samples.

	Wild/Captive Number of Elephants Sampled		Sample Number	Age	Sex	Origin	Year of Import or Rescue	EEHV1		EEHV4	EEHV5
								1A	1B		
1	Wild (*n* = 126)		W025	Sub-adult	NA *	Pu’er-Mengyang	NA	− ^$^	CN04	−	−
2			W029	Adult	NA	Pu’er-Mengyang	NA	−	CN04	−	−
3			W030	Sub-adult	NA	Pu’er-Mengyang	NA	−	CN04	−	−
4			W032	Sub-adult	NA	Pu’er-Mengyang	NA	−	CN04	−	−
5			W106	Sub-adult	NA	Shangyong	NA	−	CN04	−	−
6			W113	Sub-adult	NA	Shangyong	NA	−	CN04	−	−
7			W118	Sub-adult	NA	Shangyong	NA	−	CN04	−	−
8			W119	Sub-adult	NA	Mengla	NA	−	−	CN06	−
9			W120	Sub-adult	NA	Mengla	NA	−	−	CN06	−
10			W121	Sub-adult	NA	Mengla	NA	−	CN05	−	−
11	Captive (*n* = 199)	Zoo (*n* = 155)	Z054	Adult	F	Myanmar	1996	−	CN04	−	−
12			Z196	Sub-adult	M	Laos	2016	−	CN04	−	−
13		Wild Elephant Valley (*n* = 44)	V36	Unweaned	F	Captive bred	NA	−	−	CN06	−
14			V37	Unweaned	F	Captive bred	NA	−	−	CN06	−
15			V38	Unweaned	M	Laos	2019	−	−	CN06	−
16			V39	Unweaned	F	Captive bred	NA	−	−	CN06	−

* NA = information not available; ^$^ − = not detected.

**Table 4:** The presence of EEHV type or strain found in China and in other countries.

Strain Name	Virus Types	Positive Elephants in This Study	Presence in Other Countries				
			Country	Strain Name	Isolation Date	Origin	GenBank Accession
CN01	EEHV1A	V33	Novel Sequence				
CN02	EEHV1A	V37	Thailand	ThE03	2008	necropsy tissue from 26 month old captive-born male elephant with fatal acute hemorrhagic disease	KT390759
			Thailand	ThE07	2012	necropsy tissue from a 2.4 year old captive-born female elephant with fatal acute hemorrhagic disease	KT447201
			USA	NAP17	2000	necropsy tissue from a 6 year old captive-born female elephant with fatal acute hemorrhagic disease	KT705221
			USA	NAP79	2016	whole blood from a 2 year old female captive elephant with acute hemorrhagic disease	MK473327
CN03	EEHV1A	V42, V44, V45	USA	NAP29	2007	necropsy tissue from a 16 month old captive-born female elephant with fatal acute hemorrhagic disease	KT705228
CN04	EEHV1B	V34, V43, Z054, Z196, W025, W029, W030, W032, W113, W116, W118	Thailand	ThE04	2009	necropsy tissue from a 2 year old captive-born female elephant with fatal acute hemorrhagic disease	KT447186
			USA	NAP38	2009	routine trunk washes from healthy adult Asian elephants	GU350763
CN05	EEHV1B	V31, W121	Novel Sequence				
CN06	EEHV4	V36, V37, V38, V39, V41, V42, V43, V44, V45, W119, W120	Thailand	ThE17	2014	necropsy tissue from a 3.7 year old captive-born female elephant with fatal acute hemorrhagic disease	KT596956
			USA	NAP69	2014.08	trunk wash from a convalescing 4.5 year old captive-born male elephant	KR781027
			USA	NAP70	2014.10	trunk wash from a convalescing 4 year old captive-born female elephant	KR781034
CN07	EEHV5	V10	USA	NAP50	2011	viremic blood from a 41 year old wild-born female elephant with mild systemic infection	JN983108
			USA	NAP51WB	2011	whole blood from an asymptomatic 4 month old captive-born female elephant	JX011010
			USA	NAP52WB	2011	whole blood from a 9 month old captive-born male elephant with mild disease symptoms	JX011011

**Figure A1:**
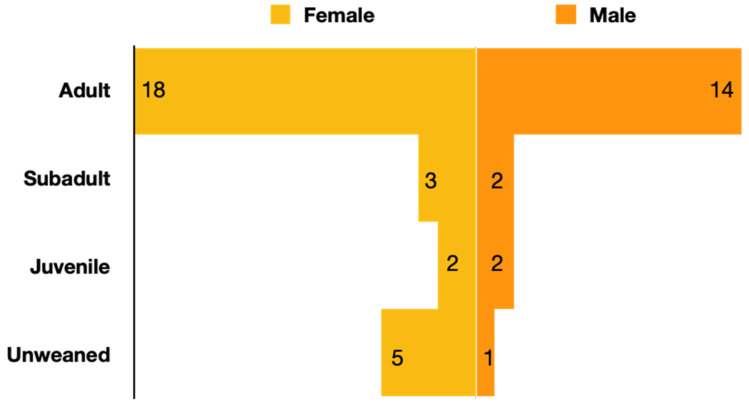
Age and sex composition of elephants in the Wild Elephant Valley.

## Data Availability

Genomic data reported in this study are available at GenBank, Accession numbers: OL799261-OL799267, OL845859.

## Appendix A

**Table A1:** Listing of EEHV primers used.

Description		Sequence (5′-3′)	Pairing and Product Size (bp)			
			Round			
			1	2A	2B	3
PANEEHV POL						
1.	6710	–ACAAACACGCTGTCRGTRTCYCCRTA–	500	250		
2.	6711	–GTATTTGATTTYGCNAGYYTGTAYCC–				
3.	6712	–TGYAAYGCCGTNTAYGGATTYACCGG–		250		
EEHV1 POL						
1.	7445	–GATTTTGCGAGYCTGTACCY–	530		500	
2.	7446	–CACGCTGTCAGTATCTCCGTA–		500		
3.	7447	–CCCAGTATCATTCAAGCATAC–				480
4.	7448	–CTGTCTACAGGGCARTCAAC–			500	
EEHV1 vGPCR						
1.	7506	–GATTGTGAACGCTGTATGTC–	930	880		
2.	4963	–GACTTTCTTCGTCGTAGCCCTCGTCTT–			700	
3.	7470	–GGTGGTACTGTATGATGTGC–				550
4.	5200	–CGTGATACGCTTCCAAACATACA–		880		
EEHV3/4 POL						
1.	6719	–CGTTGAAGGTGTCGCAGAT–	390	250		
2.	7400	–CAGCATCATCCAGGCCTACAAC–				
3.	6720	–ATCCTGGCGCAGCTGCTGAC–		250		
EEHV5 POL						
1.	7483	–CTACATCTATACAGAACTTTCC–	450		200	
2.	7484	–GTACCTTAGTTACGGACGAGAC–		430		
3.	7424	–CGACAGCACACTCCTTTAACAC–				190
4.	7485	–CGCTGTATATGGATTTACCGG–			200	
